# Supplementary material for: Assessing the diversity of whiteflies infesting cassava in Brazil
Source: PeerJ. 2021 Jul 15;9:e11741. doi: 10.7717/peerj.11741 (PMC8286705; doi:10.7717/peerj.11741)
Supplement: Supplemental Information 1 [file peerj-09-11741-s001.docx]

**Supplementary Table S1.** Number and locations of samples used for begomovirus detection.

| **Sample^a^** | **Location, state** | **Number of plants analyzed** | **Begomovirus-like symptoms** | **PCR result** | **RCA result** |
| --- | --- | --- | --- | --- | --- |
| BA2 | Luis Eduardo Magalhães, BA | 1 | nd | negative | nd |
| DF1 | Planaltina, DF | 1 | nd | negative | nd |
| DF2 | Planaltina, DF | 1 | nd | negative | nd |
| DF3 | Planaltina, DF | 1 | nd | negative | nd |
| MG1 | Ouro Fino, MG | 10 | no symptoms | negative | negative |
| MG10 | Florestal, MG | 10 | no symptoms | negative | negative^b^ |
| MG12 | Divinópolis, MG | 5 | no symptoms | negative | nd |
| MG13 | Viçosa, MG | 10 | no symptoms | negative | nd |
| MG19 | Caparaó, MG | 8 | no symptoms | negative | nd |
| MG2 | Pouso Alegre, MG | 10 | no symptoms | negative | negative |
| MG3 | Careaçu, MG | 5 | no symptoms | negative | nd |
| MG4 | Lambari, MG | 5 | no symptoms | negative | nd |
| MG5 | Lima Duarte, MG | 5 | no symptoms | negative | nd |
| MG6 | RioPomba, MG | 5 | no symptoms | negative | nd |
| MG7 | Florestal, MG | 5 | no symptoms | negative | nd |
| MG8 | Florestal, MG | 8 | no symptoms | negative | negative |
| MG9 | Florestal, MG | 5 | no symptoms | negative | nd |
| MT1 | Canarana, MT | 10 | no symptoms | negative | nd |
| MT2 | Canarana, MT | 1 | no symptoms | negative | nd |
| MT4 | Pedra Preta, MT | 1 | nd | negative | nd |
| MT5 | Pedra Preta, MT | 1 | nd | negative | nd |
| MT6 | Pedra Preta, MT | 1 | nd | negative | nd |
| PA1 | Brasil Novo, PA | 10 | no symptoms | negative | nd |
| PA2 | Vitória do Xingu, PA | 8 | no symptoms | negative | nd |
| PA3 | Altamira, PA | 8 | no symptoms | negative | nd |
| PA4 | Altamira, PA | 2 | no symptoms | negative | nd |
| PR4 | Sertanópolis, PR | 2 | nd | negative | nd |
| SP11 | Oleo, SP | 2 | nd | negative | nd |
| SP9 | Casa Branca, SP | 2 | nd | negative | nd |
| **Total** |  | 143 |  |  |  |

^a^ For detailed information about samples see Table 1*.* AL, Alagoas; BA, Bahia; DF, Federal District; ES, Espírito Santo; GO, Goiás; MG, Minas Gerais; MT, Mato Grosso; PA, Pará; PI, Piauí; PR, Paraná; SC, Santa Catarina; SP, São Paulo. nd: not determined.

^b^ RCA performed in samples MG10-5 and MG10-6.
