# Supplementary material for: Assessing the diversity of whiteflies infesting cassava in Brazil
Source: PeerJ. 2021 Jul 15;9:e11741. doi: 10.7717/peerj.11741 (PMC8286705; doi:10.7717/peerj.11741)
Supplement: Supplemental Information 2 [file peerj-09-11741-s002.docx]

**Supplementary Table S2.** Whitefly isolates obtained in this study.

| **Sample^a^** | **Collection date** | **Host** | **Species** | **Isolate^b^** | **GenBank access no.** |
| --- | --- | --- | --- | --- | --- |
| MG1 | May-18 | *Manihot esculenta* | *Tetraleurodes acaciae* | AMG1-7 | MT901081 |
| MG1 | May-18 | *Manihot esculenta* | *Tetraleurodes acaciae* | NMG1-1 | MT901082 |
| MG2 | Jul-18 | *Manihot esculenta* | *Tetraleurodes acaciae* | AMG2-1 | MT901083 |
| MG2 | Jul-18 | *Manihot esculenta* | *Tetraleurodes acaciae* | NMG2-1 | MT901084 |
| MG3 | Feb-18 | *Manihot esculenta* | *Tetraleurodes acaciae* | AMG3-2 | MT901085 |
| MG3 | Feb-18 | *Manihot esculenta* | *Tetraleurodes acaciae* | NMG3-3 | MT901086 |
| MG4 | Jun-18 | *Manihot esculenta* | *Tetraleurodes acaciae* | AMG4-2 | MT901087 |
| MG4 | Jun-18 | *Manihot esculenta* | *Tetraleurodes acaciae* | NMG4-2 | MT901088 |
| MG5 | Feb-18 | *Manihot esculenta* | *Tetraleurodes acaciae* | AMG5-11 | MT901089 |
| MG5 | Feb-18 | *Manihot esculenta* | *Tetraleurodes acaciae* | NMG5-1 | MT901090 |
| MG7 | Feb-18 | *Manihot esculenta* | *Tetraleurodes acaciae* | AMG7-1 | MT901091 |
| MG7 | Feb-18 | *Manihot esculenta* | *Tetraleurodes acaciae* | NMG7-1 | MT901092 |
| MG8 | Feb-18 | *Manihot esculenta* | *Tetraleurodes acaciae* | AMG8-1 | MT901093 |
| MG9 | Mar-18 | *Manihot esculenta* | *Tetraleurodes acaciae* | AMG9-1 | MT901094 |
| MG9 | Mar-18 | *Manihot esculenta* | *Tetraleurodes acaciae* | NMG9-1 | MT901095 |
| MG10 | Feb-18 | *Manihot esculenta* | *Tetraleurodes acaciae* | AMG10-1 | MT901096 |
| MG10 | Feb-18 | *Manihot esculenta* | *Tetraleurodes acaciae* | NMG10-1 | MT901097 |
| MG11 | Mar-18 | *Manihot esculenta* | *Tetraleurodes acaciae* | AMG11-1 | MT901098 |
| MG11 | Mar-18 | *Manihot esculenta* | *Tetraleurodes acaciae* | AMG11-8 | MT901099 |
| MG11 | Mar-18 | *Manihot esculenta* | *Tetraleurodes acaciae* | NMG11-1 | MT901100 |
| MG12 | May-18 | *Manihot esculenta* | *Tetraleurodes acaciae* | AMG12-7 | MT901101 |
| MG12 | May-18 | *Manihot esculenta* | *Tetraleurodes acaciae* | NMG12-1 | MT901102 |
| MG14 | Mar-18 | *Manihot esculenta* | *Tetraleurodes acaciae* | AMG14-5 | MT901103 |
| MG15 | Mar-18 | *Manihot esculenta* | *Tetraleurodes acaciae* | AMG15-1 | MT901104 |
| MG15 | Mar-18 | *Manihot esculenta* | *Tetraleurodes acaciae* | NMG15-2 | MT901105 |
| MG16 | Mar-18 | *Manihot esculenta* | *Tetraleurodes acaciae* | AMG16-1 | MT901106 |
| MG17 | Mar-18 | *Manihot esculenta* | *Tetraleurodes acaciae* | AMG17-9 | MT901107 |
| MG18 | Mar-18 | *Manihot esculenta* | *Tetraleurodes acaciae* | AMG18-5 | MT901108 |
| MG19 | Feb-18 | *Manihot esculenta* | *Tetraleurodes acaciae* | AMG19-10 | MT901109 |
| MG19 | Feb-18 | *Manihot esculenta* | *Tetraleurodes acaciae* | NMG19-4 | MT901110 |
| ES1 | Jan-18 | *Manihot esculenta* | *Tetraleurodes acaciae* | AES1-11 | MT901111 |
| ES1 | Jan-18 | *Manihot esculenta* | *Tetraleurodes acaciae* | NES1-1 | MT901112 |
| ES2 | Jan-18 | *Manihot esculenta* | *Tetraleurodes acaciae* | AES2-2 | MT901113 |
| ES2 | Jan-18 | *Manihot esculenta* | *Tetraleurodes acaciae* | NES2-1 | MT901114 |
| ES2 | Jan-18 | *Manihot esculenta* | *Tetraleurodes acaciae* | NES2-12 | MT901115 |
| ES3 | Jan-18 | *Manihot esculenta* | *Tetraleurodes acaciae* | AES3-2 | MT901116 |
| ES3 | Jan-18 | *Manihot esculenta* | *Tetraleurodes acaciae* | NES3-1 | MT901117 |
| PA1 | Jan-18 | *Manihot esculenta* | *Tetraleurodes acaciae* | APA1-1 | MT901118 |
| PA1 | Jan-18 | *Manihot esculenta* | *Tetraleurodes acaciae* | APA1-11 | MT901119 |
| PA1 | Jan-18 | *Manihot esculenta* | *Tetraleurodes acaciae* | NPA1-11 | MT901120 |
| PA2 | Jan-18 | *Manihot esculenta* | *Tetraleurodes acaciae* | APA2-16 | MT901121 |
| PA2 | Jan-18 | *Manihot esculenta* | *Tetraleurodes acaciae* | NPA2-2 | MT901122 |
| PA3 | Aug-18 | *Manihot esculenta* | *Tetraleurodes acaciae* | APA3-6 | MT901123 |
| PA3 | Aug-18 | *Manihot esculenta* | *Tetraleurodes acaciae* | NPA3-2 | MT901124 |
| PA4 | Jan-18 | *Manihot esculenta* | *Tetraleurodes acaciae* | NPA4-16 | MT901125 |
| AL1 | Jul-18 | *Manihot esculenta* | *Tetraleurodes acaciae* | NAL1-17 | MT901126 |
| AL2 | Apr-18 | *Manihot esculenta* | *Tetraleurodes acaciae* | AAL2-10 | MT901127 |
| AL3 | Apr-18 | *Manihot esculenta* | *Tetraleurodes acaciae* | AAL3-6 | MT901128 |
| AL4 | Apr-18 | *Manihot esculenta* | *Tetraleurodes acaciae* | AAL4-2 | MT901129 |
| AL4 | Apr-18 | *Manihot esculenta* | *Tetraleurodes acaciae* | NAL4-7 | MT901130 |
| AL5 | Apr-18 | *Manihot esculenta* | *Tetraleurodes acaciae* | NAL5-4 | MT901131 |
| BA1 | Dec-17 | *Manihot esculenta* | *Tetraleurodes acaciae* | ABA1-8 | MT901132 |
| PI1 | Apr-18 | *Manihot esculenta* | *Tetraleurodes acaciae* | NPI1-19 | MT901133 |
| BA1 | Jul-18 | *Manihot esculenta* | *Bemisia tuberculata* | AMG2-9 | MT901134 |
| MG3 | Feb-18 | *Manihot esculenta* | *Bemisia tuberculata* | AMG3-1 | MT901135 |
| MG3 | Feb-18 | *Manihot esculenta* | *Bemisia tuberculata* | NMG3-1 | MT901136 |
| MG6 | Apr-18 | *Manihot esculenta* | *Bemisia tuberculata* | AMG6-6 | MT901137 |
| MG10 | Feb-18 | *Manihot esculenta* | *Bemisia tuberculata* | AMG10-7 | MT901138 |
| MG10 | Feb-18 | *Manihot esculenta* | *Bemisia tuberculata* | NMG10-12 | MT901139 |
| MG12 | May-18 | *Manihot esculenta* | *Bemisia tuberculata* | AMG12-3 | MT901140 |
| MG13 | Aug-18 | *Manihot esculenta* | *Bemisia tuberculata* | AMG13-3 | MT901141 |
| MG15 | Mar-18 | *Manihot esculenta* | *Bemisia tuberculata* | NMG15-1 | MT901142 |
| MG16 | Mar-18 | *Manihot esculenta* | *Bemisia tuberculata* | AMG16-4 | MT901143 |
| MG18 | Mar-18 | *Manihot esculenta* | *Bemisia tuberculata* | AMG18-18 | MT901144 |
| MT1 | Dec-17 | *Manihot esculenta* | *Bemisia tuberculata* | AMT1-16 | MT901145 |
| MT1 | Dec-17 | *Manihot esculenta* | *Bemisia tuberculata* | NMT1-16 | MT901146 |
| MT2 | Dec-17 | *Manihot esculenta* | *Bemisia tuberculata* | NMT2-4 | MT901147 |
| ES3 | Jan-18 | *Manihot esculenta* | *Bemisia tuberculata* | AES3-4 | MT901148 |
| ES3 | Jan-18 | *Manihot esculenta* | *Bemisia tuberculata* | NES3-5 | MT901149 |
| PA3 | Aug-18 | *Manihot esculenta* | *Bemisia tuberculata* | APA3-7 | MT901150 |
| PA4 | Jan-18 | *Manihot esculenta* | *Bemisia tuberculata* | APA4-1 | MT901151 |
| PR1 | Mar-18 | *Manihot esculenta* | *Bemisia tuberculata* | APR1-16 | MT901152 |
| PR2 | Mar-18 | *Manihot esculenta* | *Bemisia tuberculata* | APR2-18 | MT901153 |
| GO2 | Mar-18 | *Manihot esculenta* | *Bemisia tuberculata* | AGO2-16 | MT901154 |
| AL1 | Jul-18 | *Manihot esculenta* | *Bemisia tuberculata* | NAL1-2 | MT901155 |
| AL2 | Apr-18 | *Manihot esculenta* | *Bemisia tuberculata* | AAL2-1 | MT901156 |
| AL2 | Apr-18 | *Manihot esculenta* | *Bemisia tuberculata* | NAL2-1 | MT901157 |
| AL3 | Apr-18 | *Manihot esculenta* | *Bemisia tuberculata* | AAL3-3 | MT901158 |
| AL3 | Apr-18 | *Manihot esculenta* | *Bemisia tuberculata* | NAL3-1 | MT901159 |
| AL4 | Apr-18 | *Manihot esculenta* | *Bemisia tuberculata* | AAL4-4 | MT901160 |
| AL4 | Apr-18 | *Manihot esculenta* | *Bemisia tuberculata* | NAL4-1 | MT901161 |
| BA1 | Dec-17 | *Manihot esculenta* | *Bemisia tuberculata* | ABA1-12 | MT901162 |
| PI1 | Apr-18 | *Manihot esculenta* | *Bemisia tuberculata* | NPI1-16 | MT901163 |
| GO1 | Mar-18 | *Manihot esculenta* | *Bemisia tabaci* MEAM1 | NGO1-1 | MT901164 |
| GO2 | Mar-18 | *Manihot esculenta* | *Bemisia tabaci* MEAM1 | AGO2-2 | MT901165 |
| GO2 | Mar-18 | *Manihot esculenta* | *Bemisia tabaci* MEAM1 | NGO2-5 | MT901166 |
| AL2 | Apr-18 | *Manihot esculenta* | *Bemisia tabaci* MEAM1 | AAL2-7 | MT901167 |
| AL3 | Apr-18 | *Manihot esculenta* | *Bemisia tabaci* MEAM1 | AAL3-8 | MT901168 |
| AL5 | Apr-18 | *Manihot esculenta* | *Bemisia tabaci* MEAM1 | AAL5-3 | MT901169 |
| BA1 | Dec-17 | *Manihot esculenta* | *Bemisia tabaci* MEAM1 | ABA1-20 | MT901170 |
| AL2 | Apr-18 | *Manihot esculenta* | *Bemisia tabaci* NW | AAL2-11 | MT901171 |
| MT1 | Dec-17 | *Manihot esculenta* | *Tetraleorodes sp.* ^c^ | AMT1-16 | MT901172 |
| MG6 | Apr-18 | *Manihot esculenta* | *Bemisia sp*. ^d^ | AMG6-16 | MT904381 |
| MG6 | Apr-18 | *Manihot esculenta* | *Bemisia sp*. ^d^ | NMG6-4 | MT904382 |

^a^ For detailed information about samples see Table 1*.*

^b^ First letter of isolate name indicates whether a given sequence was obtained from adults (A) or nymphs (N), followed by two letters indicating the state from where the sample was collected: AL, Alagoas; BA, Bahia; DF, Federal District; ES, Espírito Santo; GO, Goiás; MG, Minas Gerais; MT, Mato Grosso; PA, Pará; PI, Piauí; PR, Paraná; SC, Santa Catarina; SP, São Paulo.

^c^ Corresponds to WtNEW1.

^d^ Corresponds to WtNEW2.
