# Supplementary material for: Assessing the diversity of whiteflies infesting cassava in Brazil
Source: PeerJ. 2021 Jul 15;9:e11741. doi: 10.7717/peerj.11741 (PMC8286705; doi:10.7717/peerj.11741)
Supplement: Supplemental Information 4 — Fig. S1 . Spearman’s rank correlation coefficient analysis comparing number of nymphs and adults for the three more abundant whiteflies species. Each dot represent a sampled field. Only fields where nymphs and adults or where only one phase was observed were included in this analysis. Scatter plot showing 95% of confidence interval (light green) are shown. Total, correspond all three specie plotted together. [file peerj-09-11741-s004.pdf]

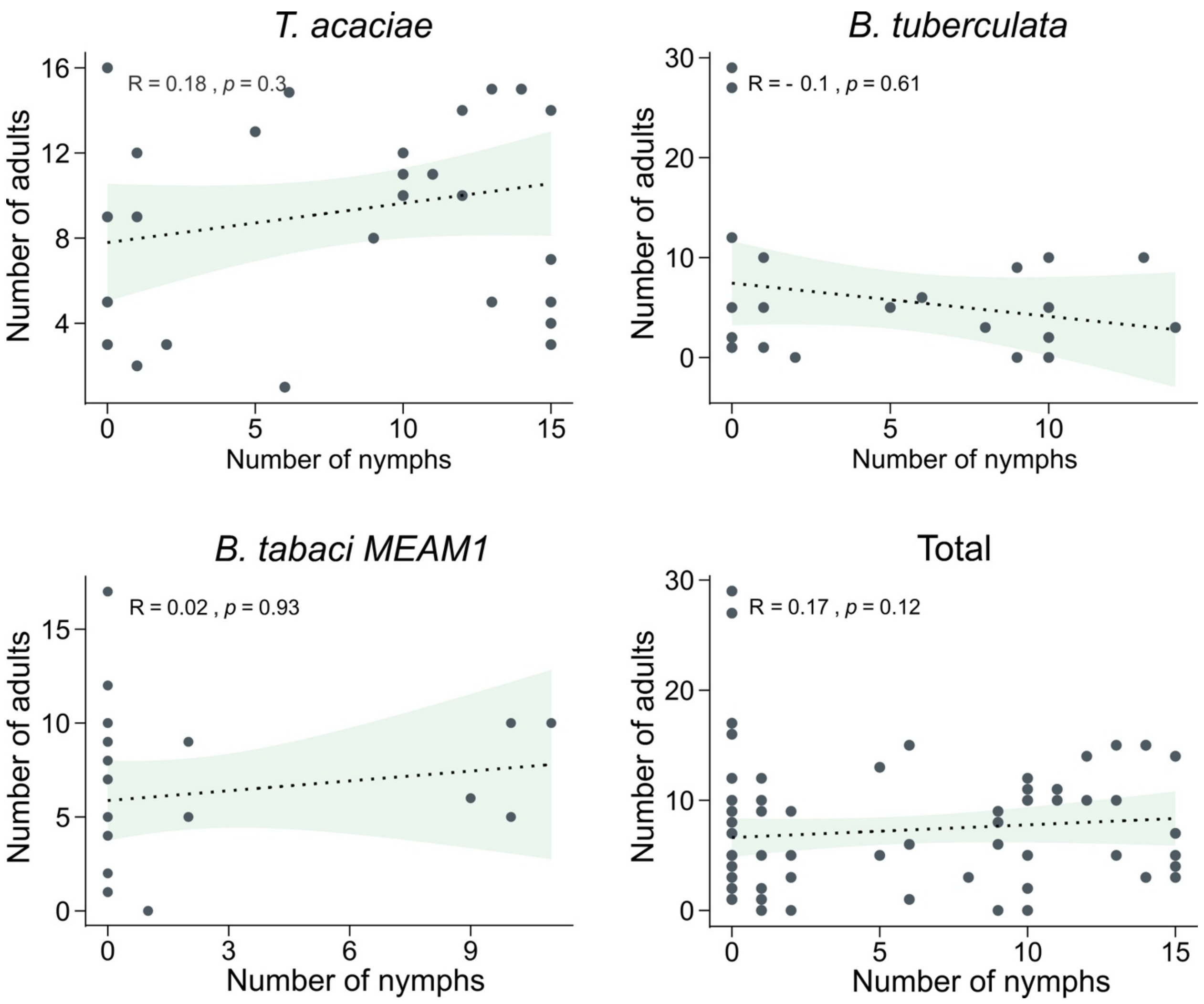

Supplementary Figure S1. Spearman's rank correlation coefficient analysis comparing number of nymphs and adults for the three more abundant whiteflies specie. Each dot represent a sampled field. Only fields where nymphs and adults or where only one phase was observed were included in this analysis. Scatter plot showing 95% of confidence interval (light green) are shown. Total, correspond all three specie ploted together.
