# Supplementary figures and images for: Assessing the diversity of whiteflies infesting cassava in Brazil
Source: PeerJ. 2021 Jul 15;9:e11741. doi: 10.7717/peerj.11741 (PMC8286705; doi:10.7717/peerj.11741)

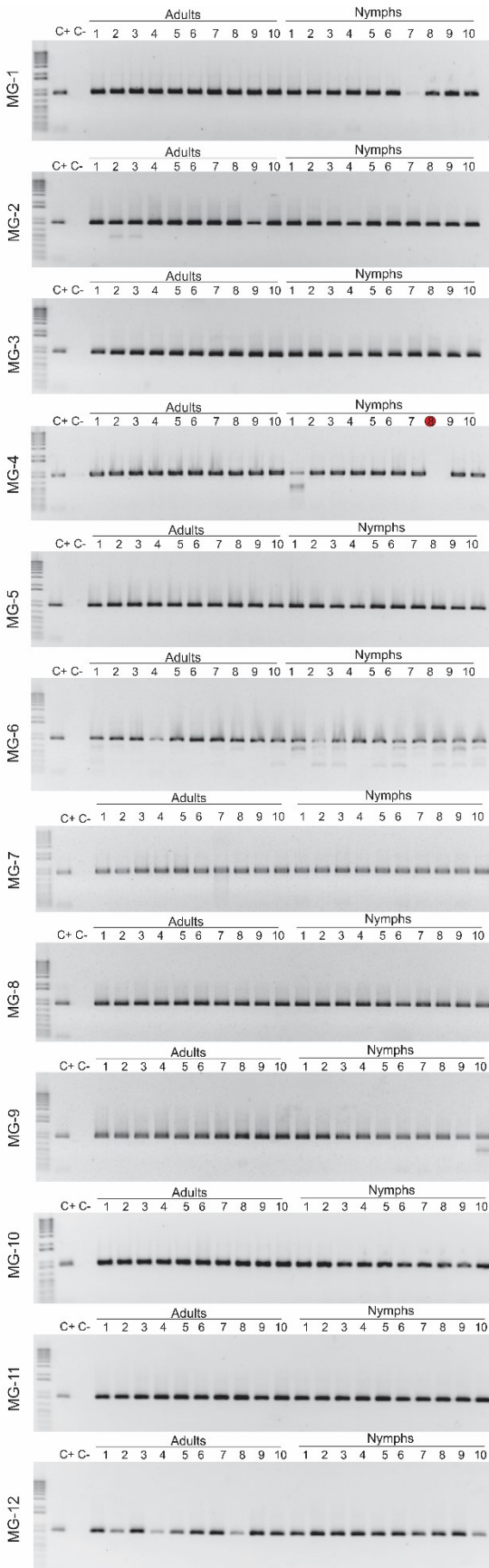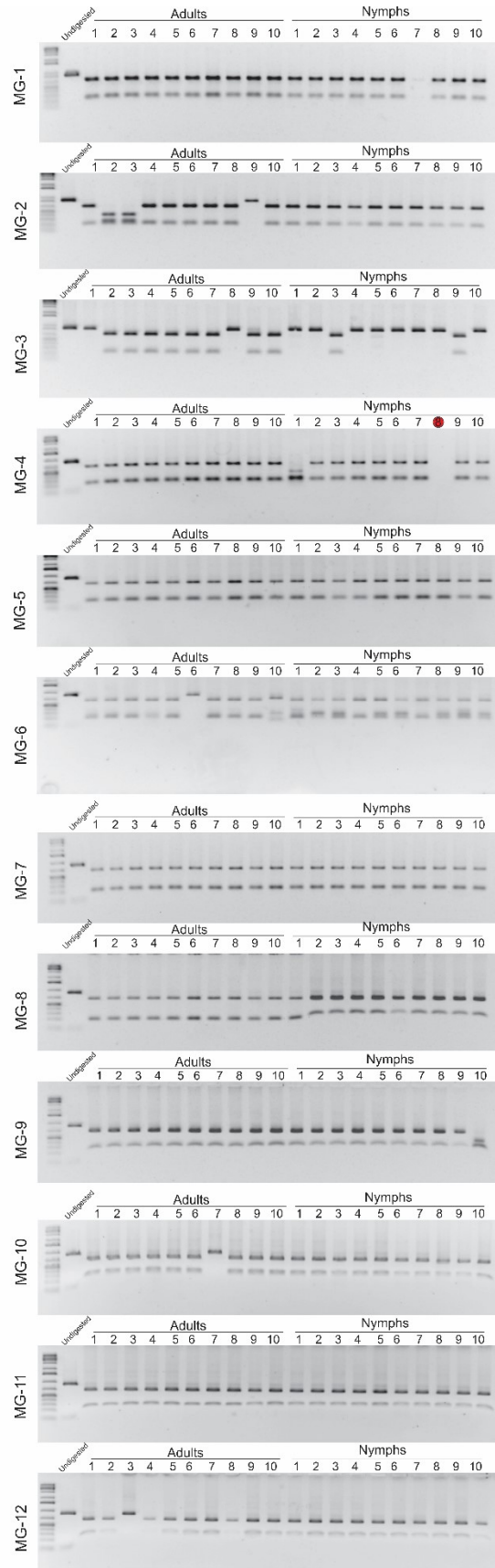

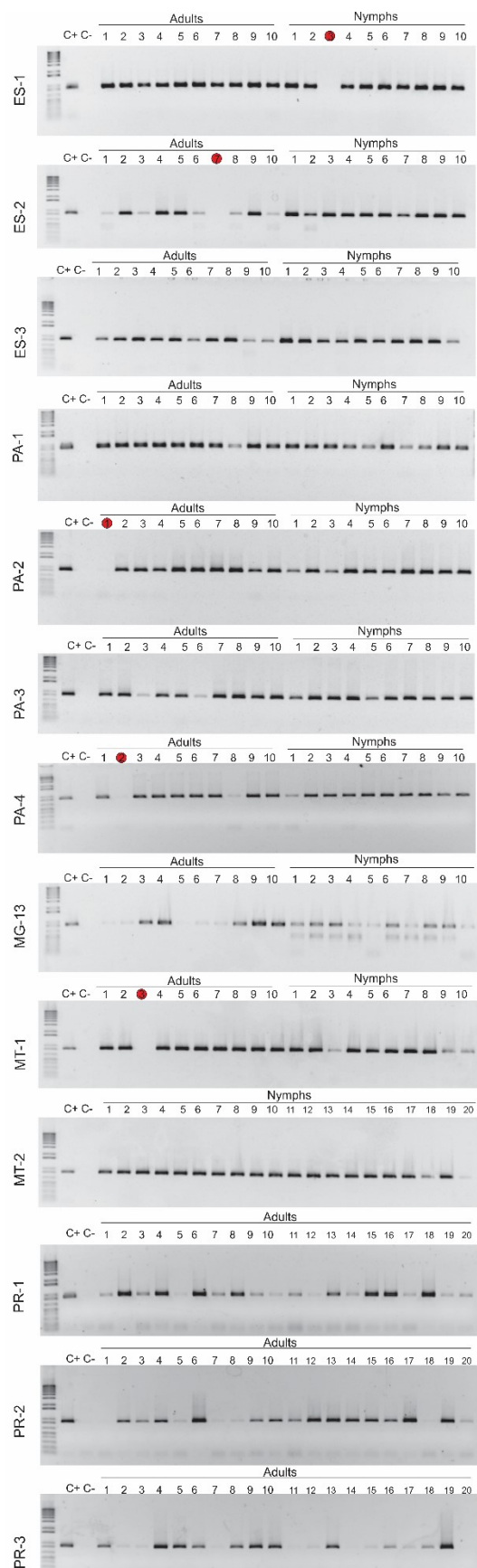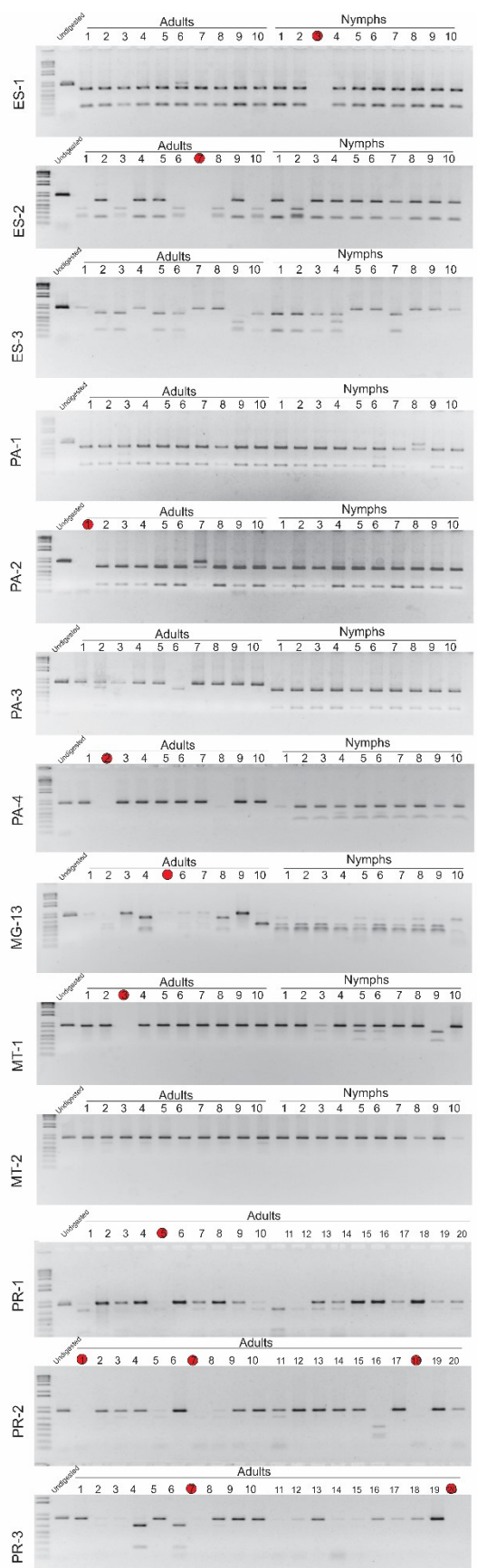

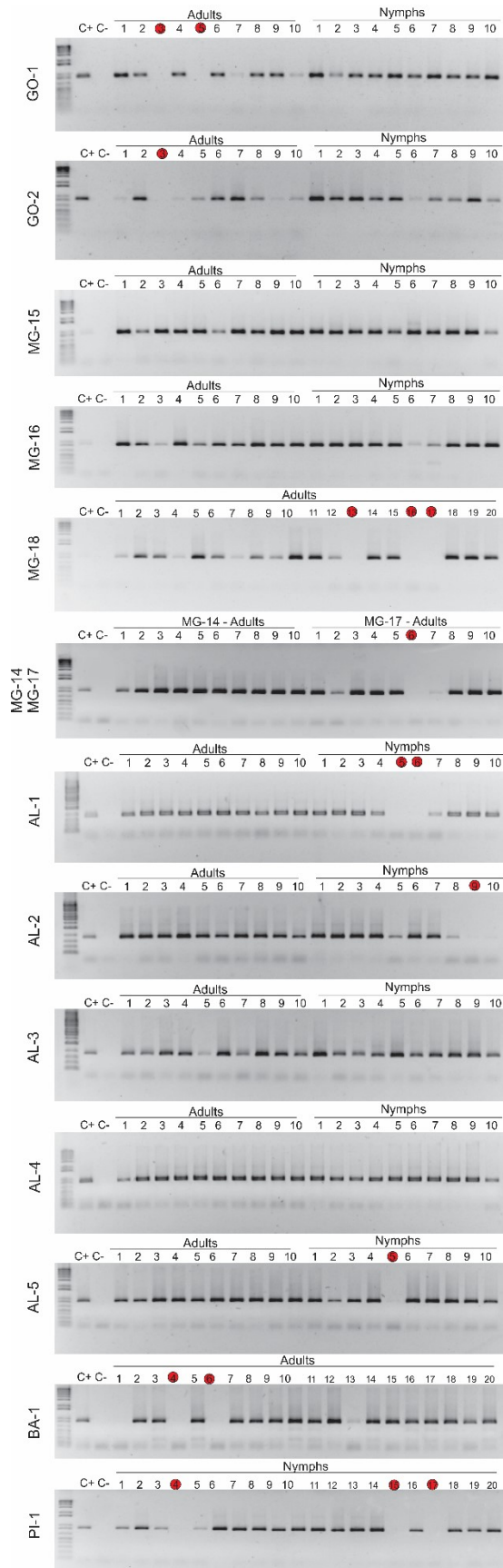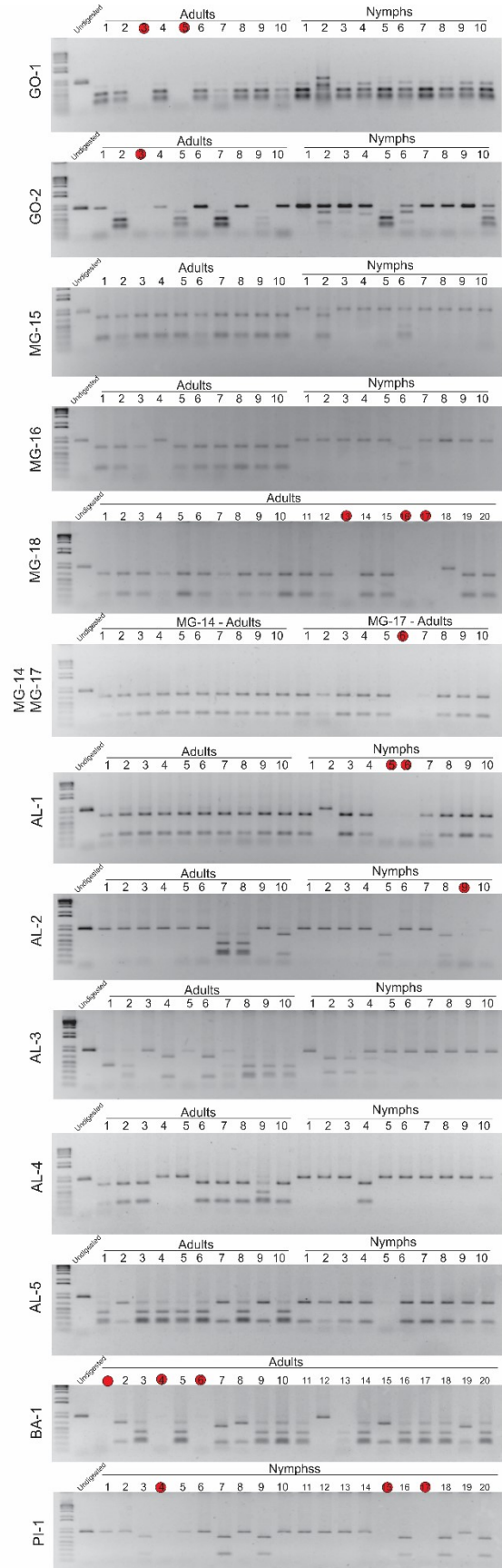

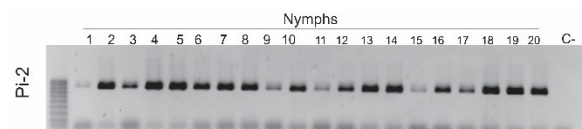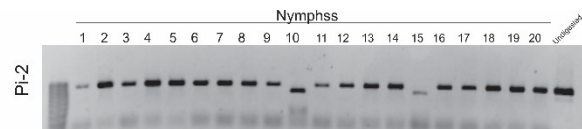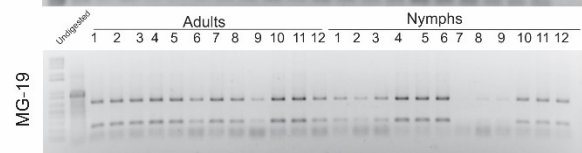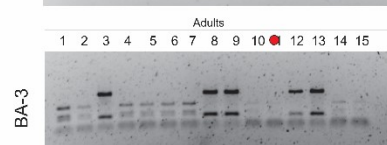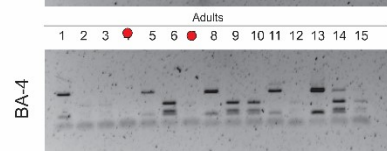

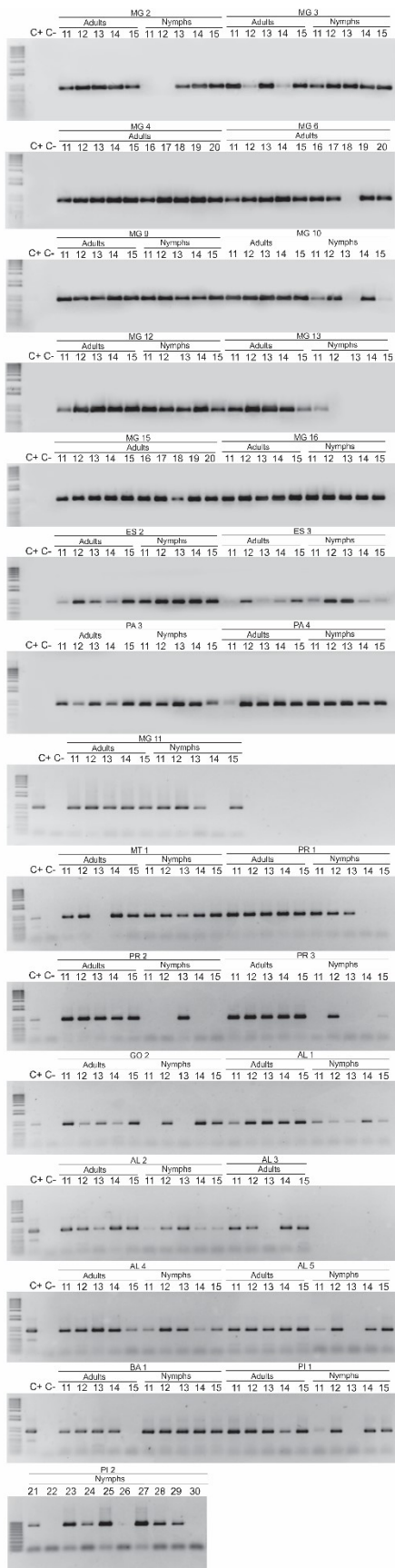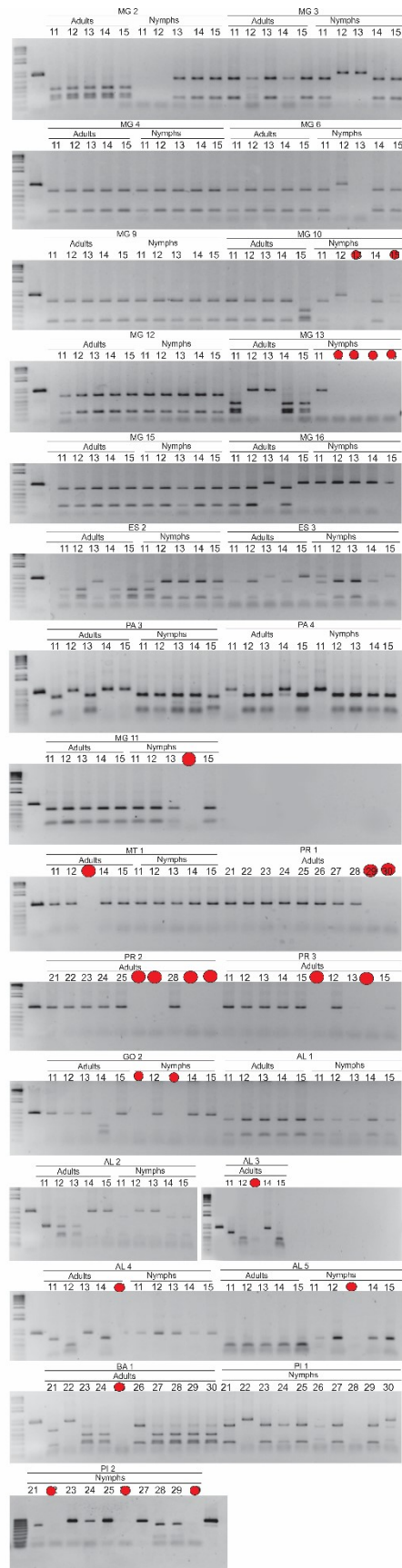

Supplement: Supplemental Information 6 — Dataset 2 (raw data). Restriction patters for the mtCOI gene used to genotype whiteflies specimens. [file peerj-09-11741-s006.pdf]
